# Biological Functions of RBP4 and Its Relevance for Human Diseases

**DOI:** 10.3389/fphys.2021.659977

**Published:** 2021-03-11

**Authors:** Julia S. Steinhoff, Achim Lass, Michael Schupp

**Affiliations:** ^1^Institute of Pharmacology, Charité-Universitätsmedizin Berlin, Corporate Member of Freie Universität Berlin, Humboldt-Universität zu Berlin, and Berlin Institute of Health, Berlin, Germany; ^2^Institute of Molecular Biosciences, NAWI Graz, University of Graz, Graz, Austria; ^3^BioTechMed-Graz, Graz, Austria

**Keywords:** RBP4, retinol transport, liver, retinoids, vitamin A, lipocalin, metabolism

## Abstract

Retinol binding protein 4 (RBP4) is a member of the lipocalin family and the major transport protein of the hydrophobic molecule retinol, also known as vitamin A, in the circulation. Expression of RBP4 is highest in the liver, where most of the body’s vitamin A reserves are stored as retinyl esters. For the mobilization of vitamin A from the liver, retinyl esters are hydrolyzed to retinol, which then binds to RBP4 in the hepatocyte. After associating with transthyretin (TTR), the retinol/RBP4/TTR complex is released into the bloodstream and delivers retinol to tissues via binding to specific membrane receptors. So far, two distinct RBP4 receptors have been identified that mediate the uptake of retinol across the cell membrane and, under specific conditions, bi-directional retinol transport. Although most of RBP4’s actions depend on its role in retinoid homeostasis, functions independent of retinol transport have been described. In this review, we summarize and discuss the recent findings on the structure, regulation, and functions of RBP4 and lay out the biological relevance of this lipocalin for human diseases.

## General Introduction

Vitamin A, which comprises retinol and its biologically relevant metabolites, belongs to the group of fat-soluble vitamins and is essential for humans (McCollum and Davis, 1913). It is famously involved in the physiology of vision, but moreover, it is also known to play roles in immune cell function, reproduction, embryonic development, and the regulation of cell proliferation and differentiation (Morriss-Kay and Sokolova, 1996; Napoli, 1996; Blaner et al., 1999; Blaner, 2007).

Most of vitamin A’s actions are mediated by its active metabolite all-*trans* retinoic acid (atRA) acting as the high affinity agonist ligand for the three known isoforms of retinoic acid receptors (RAR) α, β, and γ (Petkovich et al., 1987). A 9-*cis* configurated RA derivative, whose exact nature and physiological context is still under investigation, activates retinoid X receptors (RXR) (Heyman et al., 1992; Levin et al., 1992; Rühl et al., 2015). Both RAR and RXR belong to the family of nuclear receptors that serve as ligand-activated transcription factors. In contrast to regulating gene expression, 11-*cis* retinaldehyde in the eye is the light-sensitive chromophore of the rhodopsin complex to allow for the visual cycle in the retina (Palczewski et al., 2000). A deficit of vitamin A and as a consequence 11-*cis* retinaldehyde is evident by impaired vision, leading to night blindness or even full blindness (Blegvad, 1924). Vitamin A deficiency due to malnutrition during pregnancy is the leading cause for visual defects in newborns in developing countries (Pirie, 1983). Thus, vitamin A is required for both development (RA- and nuclear receptor-mediated) and functionality (11-*cis* retinaldehyde-mediated) of the eye, underlining its extraordinary dependence on vitamin A.

Lipocalins are a family of proteins with the ability to bind and transport small lipophilic proteins (Pervaiz and Brew, 1987). A prominent member of this family is retinol binding protein 4 (RBP4, also known as RBP) (Kanai et al., 1968; Quadro et al., 1999), which is central to this review. As suggested by its name, RBP4 transports retinol and is considered the only specific binding protein in the circulation (Kanai et al., 1968). As such and most likely also via non-canonical functions, RBP4 is implicated in a variety of human conditions that include impaired vision and ocular diseases (Li et al., 2010), disorders of glucose and lipid homeostasis (Yang et al., 2005), and cardiovascular diseases (Sun et al., 2013). This review will summarize the current knowledge of the biological functions of RBP4 and how the lipocalin is linked to these pathologies.

## Discovery and Protein Structure of RBP4

In 1968 Kanai et al. were first to describe RBP4 as a human plasma protein that is bound specifically to retinol and functioning as its transporter in the circulation. In their study, they analyzed plasma of subjects that had been injected intravenously with radio-labeled retinol. They were able to purify the protein that had bound labeled retinol and named it RBP (Kanai et al., 1968). Besides binding retinol, they found it to be circulating in complex with another, larger protein with prealbumin mobility on electrophoresis. Since then, there have been numerous studies on the biology of RBP4, dissecting its structure, function, and its role in the context of human diseases.

The primary structure of the 21 kDa protein was characterized as a single polypeptide chain, containing 201 amino acids and three disulfide bridges in humans (Rask et al., 1980, 1987). An N-terminal secretory signal peptide of 18 amino acid is cleaved upon protein processing. The complete 3D structure of RBP4 was reported by Newcomer et al. (1984). Using x-ray crystallography, they found that its structure is built not only of an N-terminal coil, a C-terminal α-helix followed by a coil region, but also a characteristic β-barrel core that they described as an eight-stranded up-and-down β-barrel. This β-barrel core is the structural part of the RBP4 molecule that is able to specifically host one molecule of retinol, which keeps this hydrophobic vitamin soluble in an aqueous milieu, and therefore capable of transporting it through the bloodstream.

## Transcriptional and Post-Translational Regulation of RBP4

RBP4 is highest expressed in the liver followed by robust expression in all adipose tissue depots (Tsutsumi et al., 1992; Wu C. et al., 2009, 2013). Nevertheless, its mRNA can be detected in several other tissues and anatomical structures, such as kidney, retinal pigment epithelium, testes, brain, lung, and the choroid plexus (Soprano et al., 1986; Blaner, 1989; MacDonald et al., 1990; Duan and Schreiber, 1992; Soprano and Blaner, 1994; O’Byrne and Blaner, 2013). Highest expression in liver coincides with the highest retinoid stores of any organ, corresponding to about 80% of all retinoids in the body (O’Byrne and Blaner, 2013).

Several genomic elements in the 5’ flanking region of the human *RBP4* gene confer its high expression in hepatocytes, although the occupancy with specific transcriptional regulators has not been characterized thoroughly (D’Onofrio et al., 1985; Colantuoni et al., 1987; Blaner, 1989). However, a multiprotein complex including high mobility group A1, protein-associated splicing factor, steroidogenic factor 1, and other proteins was shown to be recruited to its promoter, especially upon stimulation by cyclic AMP (Bianconcini et al., 2009; Chiefari et al., 2009), a known inducer of *Rbp4* mRNA expression in murine hepatocytes (Jessen and Satre, 1998). In mice, liver *Rbp4* mRNA is elevated by injecting glucagon (Bianconcini et al., 2009). Consistently, *Rbp4* mRNA expression in liver is induced by fasting. Interestingly, this fasting induction was also observed in mice that lack peroxisome proliferator-activated receptor α (PPARα) (Smati et al., 2020), the master regulator of the transcriptional response to fasting in liver (Kersten et al., 1999), which is in accordance with a cAMP-dependent and PPARα-independent mechanism.

Rats with either normal, retinol-depleted, or retinol-repleted status did not show any alterations in the expression or synthesis of *Rbp4* mRNA and protein in liver, respectively (Soprano et al., 1982), suggesting that hepatic gene expression of *Rbp4* is independent of the overall vitamin A status. On the other hand, both atRA and 9-*cis* RA induced *Rbp4* mRNA expression in murine Hepa 1-6 cells and in mouse liver *in vivo* in a dose- and time-dependent manner (Jessen and Satre, 2000), showing that at least at higher concentrations, these retinoids are able to induce *Rbp4* mRNA. Another study showed that atRA treatment of mice reduced mRNA expression of *Rbp4* in adipose tissues but not liver, whereas protein levels of RBP4 were reduced in liver and increased in serum (Mercader et al., 2008).

**FIGURE 1:**
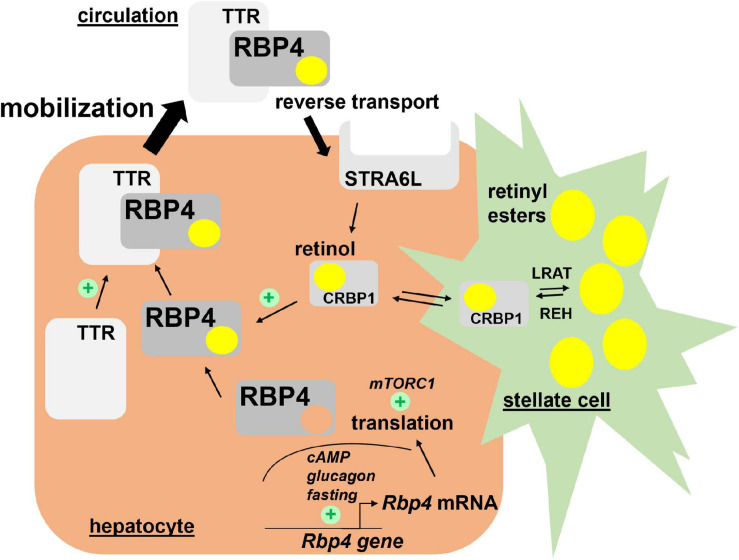
Hepatic retinol mobilization by RBP4. Most of liver retinoids are stored as retinyl esters in hepatic stellate cells in a LRAT-dependent manner. Upon hydrolysis by REH, retinol binds CRBP1 and becomes available to associate with RBP4 in hepatocytes. After complex formation with TTR, retinol/RBP4/TTR is secreted into the circulation. Both steps enhance secretion of the complex since depletion of retinol or TTR induces RBP4 accumulation in hepatocytes. Hepatocyte-expressed STRA6L is thought to confer reverse retinol transport from circulating holo-RBP4. Expression of *Rbp4* mRNA is induced by cAMP/glucagon signaling and RBP4 protein translation enhanced by mTORC1 activation. Please note that pathways for uptake and storage of dietary retinoids from circulating lipoproteins, which are thought to be RBP4-independent, are not included in the figure. cAMP, cyclic adenosine monophosphate; CRBP1, cellular retinol binding protein 1; LRAT, lecithin retinol acyltransferase; mTORC1, mechanistic target of rapamycin in complex 1; RBP4, retinol binding protein 4; REH, retinyl ester hydrolases; STRA6L, stimulated by retinoic acid 6-like; TTR, transthyretin.

Very little is known about the translational control of *Rbp4* mRNA. Welles et al. showed that in response to re-feeding of fasted mice, translation of *Rbp4* mRNA in liver was enhanced, most likely in a ‘mechanistic target of rapamycin in complex 1’ (mTORC1)-dependent manner. In support of this was the finding that rapamycin, an inhibitor of mTORC1, prevented the nutrient-induced translation of *Rbp4* mRNA (Welles et al., 2020). Major regulators of *Rbp4* transcription and translation are depicted in Figure 1.

In humans, RBP4 circulates as the native full-length protein of 183 amino acids. Interestingly, in patients with chronic renal failure, two additional forms of RBP were identified, lacking one or both of the C-terminal amino acids leucine (Jaconi et al., 1995). The authors suggested that these C-terminally truncated RBP4 proteins are generated in hepatocytes and, after its release, are rapidly cleared by the kidney in healthy individuals, but not in patients with chronic renal failure (Jaconi et al., 1995). Moreover, chronic diseases of the kidney but not of the liver in human patients associated with higher levels of truncated proteins in the circulation (Frey et al., 2008). The physiologic role of these truncated RBP4 proteins, which are still able to bind retinol (Jaconi et al., 1996), is currently unknown.

## Tissue-Specific Contributions to Circulating RBP4

The concentration of RBP4 in blood, and therefore also retinol, is rather tightly regulated and kept normally at about 2–3 μM in humans and around 1 μM in mice, despite changes in the daily uptake of retinoids with the diet (Shirakami et al., 2012). Since RBP4 is expressed in many tissues and cell types, the specific contribution of each of these sites to circulating RBP4 is of great interest for understanding its biological function. In particular its aforementioned robust expression in adipose tissue and the observed correlation to serum levels in a variety of metabolic conditions (Yang et al., 2005) sparked the idea that adipose tissue may significantly contribute to blood RBP4 (Muoio and Newgard, 2005; Tamori et al., 2006).

The site of origin was elegantly addressed by a recent study, reporting that circulating RBP4 derives exclusively from hepatocytes. This was concluded from the observation that it was undetectable in blood of mice with a hepatocyte-specific RBP4 knockout (Thompson et al., 2017). Thus, RBP4 should be considered primarily a hepatokine rather than an adipokine, which is further supported by the finding that a modest liver-specific overexpression of RBP4 readily translates in increased serum levels (Muenzner et al., 2013; Fedders et al., 2018) and that chronic liver diseases in humans that interfere with the hepatic biosynthetic capacity lead to lower RBP4 in serum (Yagmur et al., 2007). Consistently, adiponectin promoter-driven overexpression of human RBP4 increased its protein levels in adipose tissue without leading to a major elevation of circulating RBP4 when mice were fed standard chow (Lee et al., 2016). Thus, in mice and most likely also in humans, probably all circulating RBP4 is liver-derived. However, it has been hypothesized that there are disease states or certain conditions that may allow extra-hepatic RBP4 to reach the circulation. The reasons for why hepatocyte-derived RBP4 reaches the circulation but not adipocyte-expressed RBP4 are currently unknown. This is even more puzzling since cultured adipocytes or fat explants readily secrete RBP4 into the culture media (Tsutsumi et al., 1992; Wei et al., 1997; Thompson et al., 2017). Notably, muscle-specific overexpression of human RBP4 rescued RBP4 levels in the circulation of RBP4-deficient mice, suggesting that also *in vivo*, non-hepatocyte expressed RBP4 can *per se* contribute to blood RBP4 (Quadro et al., 2002).

Besides the circulation, RBP4 is also found in other compartments such as the cerebrospinal fluid (CSF), most likely by its secretion from choroid plexus cells of the blood brain barrier (MacDonald et al., 1990; Duan and Schreiber, 1992).

It should be noted that not all commercially available kits quantify RBP4 reproducibly. When comparing different methods, including sandwich enzyme-linked immunosorbent assays and competitive enzyme-linked immunoassays, quantitative western blotting standardized to full-length RBP4 came out as the superior method for measuring RBP4 in serum (Graham et al., 2007).

## Hepatic Retinol Mobilization by RBP4

Upon dietary uptake, retinoids, in form of retinyl esters, are together with other lipids packed into chylomicrons, and then released from the intestine into the lymphatic system (Goodman et al., 1965; Vogel et al., 1999). By the action of membrane-bound lipoprotein lipase (LPL), cells hydrolyze triglycerides and retinyl esters from these lipoproteins to meet their specific demands. Especially when RBP4’s function is compromised, LPL-mediated uptake of lipoprotein-derived retinyl esters can be a significant alternative source for cellular retinoids (Quadro et al., 2004). Chylomicron remnants that are formed are then taken up by the liver which serves as the main site of retinoid storage. An alternative dietary source of retinoids are provitamin A carotenoids, whose transport, cellular uptake, and metabolism differs from these pathways and which are not the focus of this article. The reader is kindly referred to other excellent reviews on this topic (von Lintig, 2012; von Lintig et al., 2020).

RBP4’s primary function is to mobilize retinol from liver (Figure 1). The liver hosts the bulk of retinoids of dietary origin as retinyl esters (Kane et al., 2008) in hepatic stellate cells, morphologically distinct and lipid droplets containing cells that are specialized in storing high concentrations of retinyl esters (Yamada et al., 1987). Stellate cells express lecithin retinol acyltransferase (LRAT), very likely the only enzyme to esterify retinol in these cells (Batten et al., 2004; Liu and Gudas, 2005; O’Byrne et al., 2005), and a number of potential lipases for retinyl ester hydrolysis to provide retinol for mobilization (Haemmerle and Lass, 2019; Wagner et al., 2020). Although it is not completely understood how retinol travels from stellate cells to hepatocytes and *vice versa*, it was shown that an absence of RBP4 did not prevent retinyl ester storage in stellate cells, implying RBP4-independent mechanisms at play (Quadro et al., 1999). For retinol mobilization into the circulation, however, RBP4 expression in hepatocytes is indeed required (Shirakami et al., 2012). Mice that lack RBP4 globally showed increased amounts of hepatic retinol and retinyl esters, whereas circulating retinol levels were decreased by almost 90% (Quadro et al., 1999). Reciprocally, an acute overexpression of RBP4 specifically in liver increased serum retinol levels with a simultaneous reduction in liver retinyl esters (Muenzner et al., 2013), underlining the pivotal role of RBP4 expression in hepatocytes for mobilizing retinol into the circulation.

Notably, residual levels of retinol in the circulation of mice lacking RBP4 show that alternative carrier proteins are present, including albumin that can readily bind retinol (Muenzner et al., 2013). Moreover, retinyl esters are also found in liver-derived very low density lipoprotein (VLDL) and LDL (Krasinski et al., 1990), suggesting that either RBP4-independent pathways for mobilization from liver exist or that retinyl esters from dietary chylomicrons can be transferred by the action of cholesteryl ester transfer protein (CETP). Collectively, these pathways seem, at least partially, to compensate when RBP4 is dysfunctional or absent (Quadro et al., 1999, 2005) and maintaining a basal degree of retinoid delivery to target cells.

Hepatocytes release RBP4 bound to retinol (holo-RBP4) and the availability of retinol facilitates its secretion (Bellovino et al., 1999). Consistently, vitamin A-deficiency in rats decreases serum-RBP4 levels while inducing its accumulation in liver (Smith et al., 1975) and adding retinol to retinol-depleted hepatocytes increases RBP4 secretion (Dixon and Goodman, 1987). Before exiting the cell, RBP4 associates with the earlier mentioned prealbumin, also known as transthyretin (TTR), a tetrameric protein of ∼55 kDa (Monaco et al., 1995; Naylor and Newcomer, 1999), forming a complex within the endoplasmic reticulum (ER) (Bellovino et al., 1996). Total lack of TTR or an acute, liver-specific depletion induced an accumulation of RBP4 in liver, suggesting that TTR enhances RBP4 secretion (Wei et al., 1995; Fedders et al., 2018). However, TTR seems not absolutely required since some RBP4 was still detectable in the circulation of TTR knockout mice and isolated hepatocytes with or without TTR expression secreted comparable amounts of RBP4 (van Bennekum et al., 2001). Moreover, despite strongly reduced serum RBP4 and retinol concentrations, TTR-deficient mice did not show any major alterations in tissue retinoid levels, including the liver and the eye (Wei et al., 1995), arguing against a limiting function of TTR in hepatic retinol mobilization and systemic homeostasis. Thus, TTR’s primary effect on RBP4 is in regard to increasing its serum half-life by formation of a higher molecular weight complex that prevents renal filtration (van Bennekum et al., 2001). Intriguingly, TTR functions also as one of the specific transport proteins for thyroid hormone (Ingbar, 1963). The potential biological interplay of retinol and thyroid hormone transport via the liver-secreted RBP/TTR complex have not been fully dissected yet.

## Receptors for RBP4

The exact nature of an earlier postulated cell-surface receptor for RBP4 remained enigmatic until 2007, when Sun et al. identified ‘stimulated by retinoic acid 6’ (STRA6) as a cell surface receptor that transports retinol provided by holo-RBP4 across the cell membrane (Kawaguchi et al., 2007). Prior to this new function, it was already known that STRA6 contains several transmembrane domains and that its expression is inducible by atRA (Taneja et al., 1995). Strikingly, STRA6 mediates bi-directional retinol transport as well as exchange between intracellular binding proteins and extracellular RBP4, thereby balancing circulating RBP4 with import and export, metabolism, and storage of intracellular retinoids and counteracting states of both retinoid insufficiency or oversupply (Kawaguchi et al., 2012). TTR partially blocks the STRA6-mediated release of retinol from holo-RBP4 and may need to dissociate from the complex before RBP4 can bind STRA6 (Kawaguchi et al., 2011). Retinol uptake by STRA6 is coupled to intracellular binding and/or storage via cellular retinol binding protein 1 (CRBP1) and LRAT activity, respectively (Isken et al., 2008; Kawaguchi et al., 2011). Accordingly, free retinol present in the membrane blocks STRA6-catalyzed retinol release (Kawaguchi et al., 2011). A site-directed mutagenesis screen identified a characteristic loop in the structure of STRA6 that is responsible for the release of retinol from its binding pocket in the β-barrel core of RBP4 (Kawaguchi et al., 2008).

**FIGURE 2:**
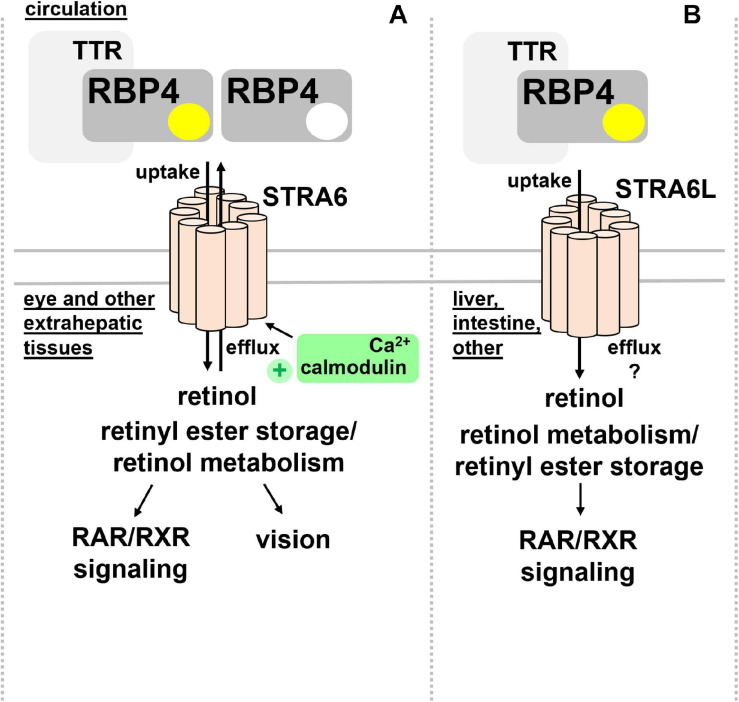
RBP4 membrane receptors for retinol transport. **(A)** Extrahepatic tissues including the eye take up retinol via binding of circulating holo-RBP4 to STRA6. Uptake is coupled to intracellular binding, storage and metabolism of retinol. STRA6 can also mediate efflux of cellular retinol to apo-RBP4, which was shown to be further stimulated by binding of calmodulin/Ca^2+^ to an intracellular domain of STRA6. **(B)** Liver, intestine, and several other organs but not the eye express STRA6L, predicted to have a similar transmembrane domain structure like STRA6. STRA6L catalyzes retinol uptake from holo-RBP4. Whether or not it can mediate bidirectional retinol transport, and how it couples to intracellular binding, storage and metabolism of retinol is currently unknown. RAR, retinoic acid receptor; RBP4, retinol binding protein 4; RXR retinoid X receptor; STRA6, stimulated by retinoic acid 6; STRA6L, stimulated by retinoic acid 6-like; TTR, transthyretin.

STRA6 is strongly expressed during embryonic development, in structures of blood-organ barriers, and organs that required high levels of retinoids, including the retinal pigment endothelium (RPE) and the female reproductive organs, testis, brain, and the kidney (Bouillet et al., 1997; Wu C. et al., 2009). In mice, differential promotor usage of the *Stra6* gene gives rise to different mRNA transcripts that show distinct regulations by retinol deficiency, potentially encoding also a shorter, N-terminally truncated STRA6 protein (Laursen et al., 2015). Mice that lack STRA6 were viable but had a dramatic reduction of retinoids in the RPE as well as retina and suffered from impaired vision and irregular ocular morphology (Ruiz et al., 2012; Amengual et al., 2014). More recently, the structure of zebrafish STRA6 was solved by cryo-electron microscopy, revealing one intramembrane and nine transmembrane helices in an intricate dimeric assembly that forms a deep lipophilic cleft for retinol internalization into the membrane lipid bilayer (Chen et al., 2016). Unexpectedly, STRA6 was tightly bound by calmodulin, which, in conjunction with Ca^2+^, was shown to favor binding of apo-RBP4 and retinol export (Zhong et al., 2020; Figure 2A). Dissecting the biological significance of calmodulin/Ca^2+^ on retinol transport by RBP4 and STRA6 may reveal an additional level of understanding of how this process is coordinated.

In addition to STRA6, a structurally related protein of about 20% overall homology acting as RBP4 receptor was identified in 2013 and designated first RBP4 receptor 2 (RBPR2) and later as ‘stimulated by retinoic acid 6-like’ (STRA6L) (Alapatt et al., 2013). Since it was known that retinol cycles between the circulation and liver and that expression of STRA6 is almost undetectable in this organ (Bouillet et al., 1997), the existence of another receptor was assumed. Indeed, STRA6L’s expression is highest in liver, and somewhat lower in intestine and colon (Alapatt et al., 2013). In contrast to STRA6, STRA6L expression is suppressed by retinol and atRA. Furthermore, it is still unknown whether STRA6L can also mediate bidirectional retinol transport (Alapatt et al., 2013; Figure 2B). Functionally, STRA6L is required for development and function of the eye in zebrafish (Shi et al., 2017; Lobo et al., 2018; Solanki et al., 2020). Also in zebrafish, STRA6L is expressed in tissues involved in retinol uptake and storage, like intestinal enterocytes, hepatocytes, and pancreatic cells but not the eye itself. However, STRA6L mutant fish exhibit visual defects that are likely due to systemic vitamin A deficiency that was observed in these fish (Shi et al., 2017).

At this point, STRA6 and STRA6L are the only known specific receptors for RBP4. It should be pointed out that retinol uptake from circulating holo-RBP4 may also involve receptor-independent membrane passage/diffusion, in particular when receptor-mediated uptake is compromised (Berry et al., 2013).

## RBP4 Catabolism

After its dissociation from TTR and retinol release, retinol-free apo-RBP4 in the circulation is filtrated by the kidney. More than 99% of that is reabsorbed by the proximal renal tubule, which renders urinary RBP4 a highly sensitive marker for tubular dysfunction (Bonventre et al., 2010). Tubular reabsorption of RBP4 is mediated by the megalin-cubilin receptor complex (Christensen et al., 1999; Raila et al., 2005). Besides its endocytic uptake, RBP4 staining in lysosomes, endoplasmic reticulum, Golgi, and basal vesicles suggest basolateral RBP4 secretion by these cells via a degradation-synthesis cycle (Christensen et al., 1999). Strikingly, kidney-specific megalin deletion in mice, besides inducing loss of RBP4 in the urine, led also to urinary retinol excretion and reduced hepatic retinol and retinyl esters, suggesting a more complex and rather unexplored role of the kidney in retinoid homeostasis (Raila et al., 2005).

## RBP4 Loss-and Gain-of-Function Induced Pathologies in Mice

**Embryonic development.** Mice with genetic deletion of RBP4 exhibit very low retinol concentrations in their circulation (Quadro et al., 1999). However, they give birth to viable embryos that show rather mild and transient developmental abnormalities of the heart (Wendler et al., 2003), a much less severe phenotype as one would have predicted from the relevance of vitamin A for the developing organism. However, this is only observed when mice were fed diets with sufficient vitamin A and implying RBP4-independent pathways for retinoid delivery already discussed above. Reducing the dietary intake of vitamin A in females lacking RBP4 before and during pregnancy has fatal consequences on embryonic development (Quadro et al., 2005). Feeding a vitamin A-deficient diet (<0.22 IU/g), severe embryonic malformations were observed, including smaller embryo size, undetectable or abnormal midfacial regions and forelimbs, and exencephaly (Quadro et al., 2005). These characteristics are in line with the severe fetal vitamin A deficiency syndrome and overlap with the phenotypes of mice with retinoid receptor knockouts (Mark et al., 2009).

**Vision.** Mice on a mixed genetic background (129xC57BL/6J) that lack RBP4 show impaired retinal function and visual acuity during the first couple of months but develop normal vision by 4–5 months of age when fed standard chow, in this particular study 22 IU of retinol/g (Quadro et al., 1999). However, when maintained on a vitamin A-deficient diet after weaning (<0.22 IU/g), vision of RBP4-deficient mice (129xC57BL/6J) further deteriorated whereas that of control mice was not compromised. A more recent study suggests that visual defects upon loss of RBP4 are even more pronounced and become chronic on a pure C57BL/6 genetic background even when feeding a vitamin A sufficient diet with ∼15 IU/g (Shen et al., 2016). Transgenic expression of human RBP4 in muscle (Quadro et al., 2002) or from the murine *RBP4* gene locus (Liu et al., 2017) rescued serum retinol levels and suppressed visual defects due to loss of endogenous RBP4. These results demonstrate that visual performance depends on RBP4-mediated retinol transport but also highlights the aforementioned existence of alternative, although less efficient, pathways for the eye to acquire retinoids (Vogel et al., 2002). It also underlines that most other tissues in the body are less dependent on RBP4-mediated retinol delivery, especially when dietary retinoid supply is ample. This notion is further supported by the phenotype of mice lacking the RBP4 receptor STRA6, in which rod photoreceptor outer and inner segment length as well as cone cell numbers were reduced, as were scotopic and photopic responses (Ruiz et al., 2012; Amengual et al., 2014). Besides visual defects, these mice showed no other major vitamin A-related abnormalities (Berry et al., 2013), somewhat similar to RBP4-deficient mice. Notably, also transgenic overexpression of human RBP4 in muscle of mice can lead to progressive retinal degeneration, although this appears to be independent of alterations in retinoids and more likely to be mediated by retinal neuroinflammation (Du et al., 2015, 2017).

**FIGURE 3:**
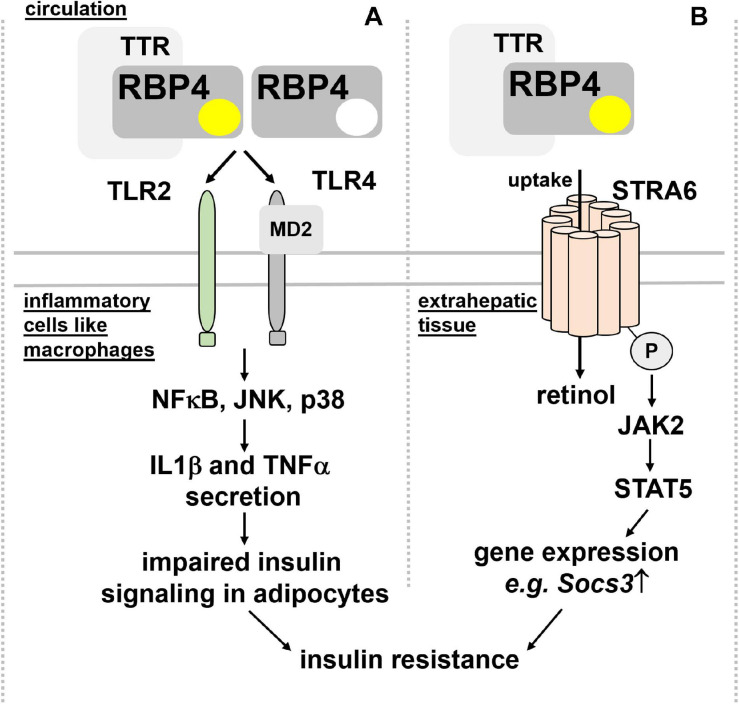
Proposed mechanisms for RBP4’s detrimental effects on insulin sensitivity. **(A)** RBP4, irrespective of its association with retinol and thus independent of retinol transport, was shown to activate TLR2/4 on inflammatory cells like macrophages. A downstream signaling cascade involving NFκB, JNK, and p38 leads to the secretion of IL1β and TNFα, which, in turn, impairs insulin signaling in adipocytes and leading to insulin resistance. **(B)** Binding of holo-RBP4 to STRA6 was shown to trigger tyrosine phosphorylation of the membrane receptor, resulting in recruitment and activation of JAK2 and the transcription factor STAT5. As a consequence, the induction of STAT5 target genes like *Socs3* impairs insulin signaling. IL1β, interleukin 1β; JAK2, Janus kinase 2; JNK, c-Jun N-terminal kinases; NFκB, nuclear factor κ-B; MD2, myeloid differentiation protein-2; RBP4, retinol binding protein 4; *Socs3*, suppressor of cytokine signaling 3; STAT5, signal transducer and activator of transcription 5; STRA6, stimulated by retinoic acid 6; TNFα, tumor necrosis factor α; TLR2/4, toll-like receptors 2/4; TTR, transthyretin.

**Insulin sensitivity and glucose tolerance.** Elevated RBP4 in the circulation of type 2 diabetic patients was reported many years ago (Basualdo et al., 1997; Abahusain et al., 1999) but it took until 2005, when a causal link between circulating RBP4 and insulin resistance was presented: Yang et al. showed, besides increased RBP4 in blood of multiple insulin resistant mouse models, that RBP4-deficient mice were less prone to develop insulin resistance (Yang et al., 2005). Moreover, transgenic overexpression of human RBP4 or injection of recombinant human RBP4 in wild-type mice caused glucose intolerance and insulin resistance. Since RBP4 expression was increased in adipose tissue but not liver, these findings led to the hypothesis that RBP4 acts as an adipokine, linking obesity with insulin resistance. Intriguingly, serum RBP4 correlated positively with adipose *RBP4* mRNA and intra-abdominal fat mass and inversely with insulin sensitivity also in humans (Klöting et al., 2007) and was lowered by exercise (Graham et al., 2006). However, at least in mice and as already pointed out above, circulating RBP4 is derived primarily from liver and not adipose tissue. Mechanistically, RBP4 was shown to induce expression of the gluconeogenic enzyme phosphoenolpyruvate carboxykinase in liver and to impair insulin signaling in muscle (Yang et al., 2005). However, follow-up studies by the same laboratory implicated the immune system, in particular antigen-presenting cells, such as dendritic cells, macrophages, and also CD4 T cells as the drivers of an inflammatory response that is induced by RBP4 within adipose tissue (Norseen et al., 2012; Moraes-Vieira et al., 2014, 2016, 2020). Strikingly, this inflammatory reaction was independent of RBP4’s association with retinol (Norseen et al., 2012) and mediated by an activation of toll-like receptors 2/4 (TLR2/4) and proinflammatory cytokine secretion from macrophages, involving nuclear factor κ-B (NFκB), c-Jun N-terminal kinases (JNK), and interleukin 1β (Moraes-Vieira et al., 2014, 2020; Figure 3A).

An alternative functional model was suggested by Berry et al. (2011), where holo-RBP4’s binding to STRA6 triggers receptor phosphorylation close to its C-terminus and the recruitment and activation of Janus kinase 2 (JAK2) and the signal transducer and activator of transcription 5 (STAT5) (Figure 3B). Interestingly, neither retinol nor retinol free apo-RBP4 were able to induce STAT5 phosphorylation. Activated STAT5 then leads to the upregulation of genes which are known to inhibit insulin signaling, such as suppressor of cytokine signaling 3 (SOCS3) (Berry et al., 2011). Follow-up studies provided evidence that activation of JAK/STAT by STRA6 requires transfer of retinol from holo-RBP to an intracellular acceptor, such as CRBP1 or LRAT (Berry et al., 2012b). TTR’s association with holo-RBP4 prevented STRA6 binding and, subsequently, induction of JAK/STAT signaling (Berry et al., 2012a). These findings were corroborated by the observation that injected recombinant holo-RBP4 failed to induce JAK/STAT or impair insulin signaling in mice lacking STRA6 (Berry et al., 2013).

Hence, RBP4’s causal role in inducing insulin resistance is still vividly debated within the field (Fedders et al., 2015). Although elevated levels of circulating RBP4 in states of insulin resistance and type 2 diabetes were reproduced by the majority of studies and by many independent laboratories, the underlying reasons for this elevation, and whether or not RBP4 is indeed actively contributing to insulin resistance is still under investigation. A few examples of unanswered issues for the reader’s consideration are described in the following sentences. For instance, not all studies identified elevated expression of RBP4 in adipose tissue of obese patients (Janke et al., 2006), and instead of adipose tissue expression, kidney function was proposed as major determinant of its serum levels, which is known to deteriorate upon the onset of type 2 diabetes and thus may lead to an accumulation of RBP4 in the blood (Raila et al., 2007; Henze et al., 2008). Other studies failed to reproduce improved insulin sensitivity and glucose tolerance in RBP4-deficient mice when fed a high-fat diet (Motani et al., 2009). Also mouse models that increase circulating RBP4 levels by an extent that is comparable to insulin resistant states by an acute or long-term liver-specific overexpression of murine RBP4 instead of the human protein did not observe an impairment in insulin responses and glucose tolerance (Muenzner et al., 2013; Fedders et al., 2018). From a physiological perspective, how can the lipocalin RBP4 that is so strongly expressed in liver and circulating at rather high levels in the blood become an inflammatory stressor upon a 2–3 fold elevation that is observed in these states? Are there yet unidentified post-translational modifications of RBP4 that occur upon extended serum half-life that could mediate this? Moreover, is retinol transport just a bystander or an active participant in these presumed RBP4 effects? Why are some of the metabolic effects of transgenically elevated RBP4 in mice seen with human but not the murine protein? Further research is needed to address these questions for building a consensus on this important, but continuously controversial topic.

**Adipose tissue lipolysis and hepatic fat content.** Adipose tissue-specific overexpression of human RBP4 in mice induces hepatic steatosis, despite an absence of changes in total RBP4 levels in the circulation or alterations in retinoid concentrations (Lee et al., 2016). Hepatic steatosis was accompanied by glucose intolerance and elevated concentrations of non-esterified fatty acids in plasma, which were shown to contribute to hepatic triglyceride accumulation. Feeding a high-fat diet aggravated the RBP4-induced metabolic disturbances in these mice. These findings indicate that locally expressed RBP4 stimulates adipose tissue lipolysis and fatty acid release, which subsequently triggers hepatic steatosis. Whether this is due to direct effects of RBP4 on fatty acid handling or indirect, via an induction of pro-inflammatory cytokines like tumor necrosis factor α (TNFα) or changes in local retinoid homeostasis, will be interesting to dissect (Lee et al., 2016). Additional insights come from another study showing that treatment of human adipocytes with RBP4 directly stimulated lipolysis and that, when co-cultured with macrophages, pro-inflammatory cytokines contribute to this by interfering with the insulin-dependent suppression of lipolysis (Kilicarslan et al., 2020). In agreement with RBP4’s stimulating effect on lipolysis are the reported lower levels of non-esterified fatty acids in serum of RBP4-deficient mice (Yang et al., 2005).

**Cold tolerance.** Exposing mice or humans to low temperatures increases plasma concentrations of RBP4 and retinol (Fenzl et al., 2020). In mice, this coincides with an elevation of *RBP4* mRNA expression in liver (Fenzl et al., 2020), which may indicate increased retinol mobilization from liver into the blood stream for supporting the adaptation to cold. Indeed, mice lacking RBP4 failed to induce thermogenic reprogramming of their subcutaneous adipose tissue, rendering these mice more sensitive to cold and dropping of their body temperature. Retinol induced thermogenic gene expression also in human white adipocytes and increased mitochondrial respiration (Fenzl et al., 2020). Thus, elevated retinol mobilization by RBP4 from liver upon the exposure to cold may be required for an adequate thermogenic adaptation of specific white adipose tissues depots in order to maintain body core temperature, which so far has not been formally tested. Moreover, loss of RBP4 in mice was associated with reduced phosphorylation of hormone sensitive lipase during the exposure to cold (Fenzl et al., 2020), a lipolytic enzyme that is activated by phosphorylation and essential for adequate fatty acid release from white adipose tissue (Stralfors and Belfrage, 1983). Providing fatty acids is required for uncoupling protein-induced thermogenesis (Schreiber et al., 2017). Thus, RBP4’s function during cold exposure may be linked to the above described stimulating effects on lipolysis, thereby ensuring fatty acid supply to brown adipose tissue as fuel for functional uncoupling protein-mediated non-shivering thermogenesis.

**Cardiovascular system and blood pressure.** Circulating RBP4 levels also correlate with blood pressure and cardiovascular disease (Solini et al., 2009; Meisinger et al., 2011; Sun et al., 2013; Li et al., 2019), potentially also secondary to decreased renal clearance due to hypertensive nephropathy (Majerczyk et al., 2017). Strikingly, systolic and diastolic blood pressure were lower in the RBP4-knockout mice and higher in the RBP4-overexpressing mice compared with the corresponding wild-type littermates (Kraus et al., 2015), suggesting indeed a functional role of RBP4 in the control of blood pressure. RBP4-deficient mice were partially protected from angiotensin 2-induced hypertension and cardiac hypertrophy. Further studies are needed to interrogate whether these effects are dependent on retinol transport and to delineate the underlying mechanisms in more detail. In this regard, effects of RBP4 on endothelial cells are likely to be involved since carbachol-induced, and therefore endothelium-dependent, vasodilation of carotid arteries *ex vivo* was slightly enhanced or reduced in RBP4 knockout and overexpressing mice, respectively (Kraus et al., 2015).

**Behavior and neuropathology.** Besides its expression in various regions of the central nervous system and associated structures (MacDonald et al., 1990; Duan and Schreiber, 1992; Wu C. et al., 2009), very little is known about RBP4’s role in the brain. Interestingly, RBP4-deficient mice have decreased locomotor activity and increased anxiety-like behavior. At a structural level these mice show neuronal loss and gliosis in the cortex and hippocampus and a reduction in proliferating neuroblasts in the subventricular zone (Buxbaum et al., 2014). It thus appears that altered vitamin A transport affects brain development and neuronal function, thereby altering behavior. Moreover, these neuropathological alterations might complicate the interpretations of other phenotypes observed in whole body RBP4-deficient mice. Of note, Buxbaum et al. investigated TTR-deficient mice in parallel and found some, but not complete overlap in the above described phenotypes, which is in accordance with the interdependent transport characteristics of the RBP/TTR complex.

## Pathologies Associated with RBP4 Mutations in Humans

So far, null mutations of RBP4 have not been characterized in humans (Zhong et al., 2014), which may suggest that in contrast to mice, complete absence of RBP4 is incompatible with embryonic survival, regardless of maternal vitamin A intake. It is interesting to note that compared to rodents, humans seem to be much more sensitive to dysregulated retinoid homeostasis and retinoid toxicity associated with random retinoid diffusion (Nau, 2001), which could be a likely consequence of dysfunctional RBP4 variants.

**Vision.** Two mutations p.I59N and p.G93D, corresponding to I41N and G75D of RBP4 after cleavage of the signal peptide of the mature protein, were associated with night blindness and modest retinal dystrophy without apparent effects on growth in compound heterozygous sisters (Biesalski et al., 1999; Seeliger et al., 1999). Both mutations were associated with reduced plasma RBP4 and retinol concentrations most likely due to reduced stability of the retinol-RBP4 complex (Folli et al., 2005). Another two missense mutations, p.A73T and p.A75T, were identified to cause autosomal dominant congenital eye malformations, including microphthalmia, anophthalmia, and coloboma (MAC) disease and with a maternal penetrance significantly greater than paternal penetrance (Chou et al., 2015). Similar to p.I59N and p.G93D, both mutant proteins bound retinol rather poorly, but strikingly, the unliganded mutant proteins were found to occupy STRA6 with much higher affinity than wild-type RBP4, and consequently are likely to disrupt delivery of vitamin A to target cells in accordance with a dominant-negative effect (Chou et al., 2015). Rare bi-allelic mutations (c.248 + 1G > A) of *RBP4* were identified in a patient with retinal dystrophy and ocular coloboma (Khan et al., 2017). A homozygous splice site variant in *RBP4* (c.111 + 1G > A) was found to cause retinal dystrophy and developmental abnormalities (Cukras et al., 2012). Both mutations were associated with low or undetectable RBP4 levels in the circulation. Another homozygous mutation (c.67 C > T) of *RBP4*, predicted to encode an early stop codon (p.Arg23^∗^) and resulting a premature termination of translation, associated with retinitis pigmentosa (Cehajic-Kapetanovic et al., 2020). Although assumed to yielding no functional RBP4, whether or not the latter mutation represents a null mutation in homozygosity is currently unclear.

It is interesting to note that mutations in the RBP4 receptor *STRA6* in humans cause a wide array of malformations including anophthalmia, congenital heart defects, diaphragmatic hernia, alveolar capillary dysplasia, lung hypoplasia, and intellectual disability, also referred to as Matthew-Wood syndrome (Golzio et al., 2007; Pasutto et al., 2007; Chassaing et al., 2009). Why the human phenotype is much more severe than STRA6 loss-of-function in mice is still under investigation but may be linked to the aforementioned differences in sensitivities to retinoid toxicity between humans and rodents.

**Other conditions.** Although also restricted to very few case studies, some of the above described RBP4 mutations were associated with acne vulgaris, osteoarthritis, and hypercholesterolemia, conditions that can be linked to dysregulated retinoid homeostasis (Seeliger et al., 1999; Cukras et al., 2012; Khan et al., 2017; Cehajic-Kapetanovic et al., 2020).

## *RBP4* Polymorphisms in Humans and Disease Association

Not unexpectedly, single-nucleotide polymorphisms of *RBP4* exist that associate with circulating retinol levels (Mondul et al., 2011). In regard to disease states, polymorphisms of *RBP4* were shown to associate with the risk for coronary artery disease (Wan et al., 2014), childhood obesity and cardiovascular risk factors (Codoñer-Franch et al., 2016), with plasma RBP4 levels and hypertriglyceridemia risk in Chinese Hans (Wu Y. et al., 2009), serum HDL (Shea et al., 2010), the risk for gestational diabetes (Hu et al., 2019), body mass index (Munkhtulga et al., 2010), insulin resistance (Kovacs et al., 2007), and type 2 diabetes (Craig et al., 2007; Munkhtulga et al., 2007; van Hoek et al., 2008). As delineated above, genetic mouse models seem to support a causative role for RBP4 in some of these conditions.

## Pharmacological Approaches to Lower Circulating RBP4

The notion that elevated RBP4 in the circulation may contribute to certain pathologies sparked renewed interest in RBP4-lowering therapies. The retinoid fenretinide, first produced in the United States in the 1960s, is a synthetic amide derivative of atRA and has antiproliferative and apoptotic effect in certain tumor cells (Ulukaya and Wood, 1999). Fenretinide was also shown to bind RBP4, to affect its hepatic secretion, and to sterically disrupt complex formation with TTR (Malpeli et al., 1996; Holven et al., 1997). As a consequence of the latter, renal clearance of RBP4 is increased and its serum concentration and also that of retinol decline (Formelli et al., 1993; Schaffer et al., 1993). This may be why in humans, fenretinide administration can lead to impaired visual adaptation to darkness (Decensi et al., 1994).

Treating high-fat diet fed mice with fenretinide for 6 weeks normalized circulating RBP4 levels to that of mice fed normal chow, at the same time improving insulin action and glucose tolerance (Yang et al., 2005), which is in support of RBP4’s detrimental effect on glucose homeostasis. Moreover, long-term treatment with fenretinide for several months prevented high-fat diet induced obesity, insulin resistance, and hepatic steatosis, whereas, unexpectedly, some of these beneficial effects were also observed in mice lacking RBP4 (Preitner et al., 2009), proposing also RBP4-independent mechanisms at play. Indeed, follow-up studies implied direct regulation of retinoid homeostasis genes by fenretinide as a likely mechanism for its metabolic benefits (McIlroy et al., 2013). Moreover, lowering RBP4 by fenretinide was dependent on the presence of LRAT, which hints toward even more complex effects of this compound (Amengual et al., 2012). Fenretinide has also been used in human trials for age-related macular degeneration with some positive outcome but limited efficacy (Mata et al., 2013). RBP4 lowering is thought to limit the accumulation of lipofuscin bisretinoids in the RPE, a process known to contribute to a variety of degenerative retinal diseases (Radu et al., 2005). However, as a retinoid, fenretinide is accompanied by a problematic safety profile including teratogenicity.

Another small molecule RBP4 ligand is A1120, which, in contrast to fenretinide, is a non-retinoid compound, bearing no similarities with atRA. A1120 disrupts the RBP4 and TTR complex by inducing a conformational change of the interaction interface and potently lowers serum RBP4 and retinol (Motani et al., 2009). In high-fat diet fed mice, both fenretinide and A1120 lowered serum RBP4 but only fenretinide improved insulin responses and glucose tolerance, which let the authors argue that fenretinide’s effects are independent of lowering RBP4 (Motani et al., 2009). By lowering retinol levels in serum and its non-retinoid nature, which may confer a more favorable safety profile compared to fenretinide, also A1120 is an investigational therapy for Stargardt macular dystrophy (Dobri et al., 2013; Hussain et al., 2018). Moreover, human RBP4 was shown to interact with engineered RBP4 protein scaffolds in an A1120-dependent manner, allowing for a synthetic and conformation-specific ON-switch system with a broad potential for pharmacological applications (Zajc et al., 2020).

BPN-14136 is another non-retinoid compound that disrupts the RBP4 and TTR complex with the potential to treat atrophic age-related macular degeneration and Stargardt disease (Cioffi et al., 2014). Interestingly, a BPN-14136 derivative was able to lower RBP4 and partially prevent high-fat diet induced obesity and hepatic steatosis in mice with adipose tissue-specific transgenic overexpression of human RBP4 described above (Cioffi et al., 2019).

## Interaction of RBP4 With Non-Retinoid Ligands

Whether endogenously expressed RBP4 transports non-retinoid ligands is currently unknown. Clues come from Nanao et al., who presented RBP4 crystal structures that contain expression host-derived oleic and linoleic acid in its ligand binding pocket, suggesting that RBP4 can indeed bind fatty acids [Protein data bank ID (Berman et al., 2000): 2WQ9 (Nanao et al., 2010), 2WR6 (Huang et al., 2010)]. Another study analyzed crystal structures of retinol-free RBP4 purified from human plasma, urine, or amniotic fluid and confirmed its binding to certain fatty acids. This included palmitic acid, which could be purified from RBP4 in human urine (Perduca et al., 2018). Moreover, palmitic and also linoleic acid, among others, were able to replace ^3^H-retinol from human RBP4 (Cioffi et al., 2019). Thus, RBP4 may be a physiologically relevant binding and transport protein for fatty acids. One could hypothesize that this may actually be involved in some of the aforementioned effects on glucose and fatty acid homeostasis and should be tested in future research.

## Concluding Remarks

Much has been learned about RBP4’s function in health and disease since its discovery more than 50 years ago. Extraordinary progress was made by generating and analyzing transgenic mouse models, in particular mice that lack RBP4 (Quadro et al., 1999), and by studying human mutations. Major findings from these studies are summarized in Table 1. What becomes obvious is that transport of retinol by RBP4 is the single most important route for its distribution in the body although, especially in mice, evolutionary pressure has led to the alternative delivery pathways to compensate for RBP4 dysfunction as a backup plan for the organism. This is also in accordance with the fundamental role of vitamin A for cell homeostasis. As pointed out in the introductory section, development of the eye and visual function show the highest dependence on the supply of retinol by RBP4, which is mirrored by the phenotypes of transgenic mouse models and the consequences of mutations in humans, and also therapeutic strategies that target this lipocalin. Besides mobilizing retinol from liver, its role within other tissues is much less well explored. Tissue-specific knockout mouse models of RBP4 may help to decipher new biological aspects. Together with potential functions beyond transporting retinol, as discussed in this review, the next decades will warrant many more exciting findings for a more complete picture of RBP4’s biology.

**TABLE 1:** Major findings from mouse models and human mutations of RBP4.

Species	Mouse model or human mutation	Findings
Mouse	Global RBP4 knockout	circulating retinol levels decreased by ∼90% (Quadro et al., 1999)increased contents of retinol and retinyl esters in liver at an age of 5 months (Quadro et al., 1999)impaired retinal function and visual acuity during first months of life that normalize after 4–5 months when fed a vitamin A-sufficient diet (Quadro et al., 1999), analyzed on mixed 129xC57BL/6J genetic backgroundviable embryos with mild and transient developmental abnormalities of the heart (Wendler et al., 2003)increased utilization of lipoprotein-derived retinyl esters (Quadro et al., 2004)increased insulin sensitivity (Yang et al., 2005)lower circulating levels of non-esterified fatty acids (Yang et al., 2005)feeding a vitamin A-reduced diet before and/or during pregnancy leads to severe embryonic malformations (smaller size, undetectable or abnormal midfacial regions and forelimbs, and exencephaly) (Quadro et al., 2005)no effect on insulin sensitivity and glucose tolerance on normal chow and after feeding a high-fat diet (Motani et al., 2009)decreased locomotor activity (Buxbaum et al., 2014)increases anxiety-like behavior (Buxbaum et al., 2014)neuronal loss (Buxbaum et al., 2014)gliosis in the cortex and hippocampus (Buxbaum et al., 2014)reduction in proliferating neuroblasts in the subventricular zone (Buxbaum et al., 2014)lower blood pressure (Kraus et al., 2015)partially protected from angiotensin 2-induced hypertension (Kraus et al., 2015)reduced cardiac hypertrophy (Kraus et al., 2015)serum retinol levels below detection threshold (Shen et al., 2016), analyzed on pure C57BL/6 genetic backgroundmore severe and persistent visual impairments (Shen et al., 2016), analyzed on pure C57BL/6 genetic backgroundimproved insulin responses and lower adipose tissue inflammation and CD4 T-cell activation when fed normal chow or high-fat diet (Moraes-Vieira et al., 2016), analyzed after feeding a low vitamin A diet (4 IU/g) for 4–5 generations prior to characterizationlower body core temperature when exposed to cold (Fenzl et al., 2020)reduced thermogenic activation and hormone sensitive lipase activity in subcutaneous white adipose tissue upon cold exposure (Fenzl et al., 2020)
Mouse	Overexpression of human RBP4 under the control of the mouse muscle creatine kinase promoter	rescue of RBP4 and retinol levels in circulation when crossed into RBP4-deficient mice (Quadro et al., 2002)suppression of visual defects when crossed into RBP4-deficient mice (Quadro et al., 2002)insulin resistant at 12 weeks of age (Yang et al., 2005)progressive retinal degeneration (Du et al., 2015)no effect on serum insulin levels or insulin sensitivity (Du et al., 2015)higher blood pressure (Kraus et al., 2015)impaired glucose tolerance and insulin sensitivity and increased adipose tissue inflammation (Moraes-Vieira et al., 2016, 2020)
Mouse	Acute liver-specific overexpression of murine RBP4	increased serum RBP4 and retinol levels and RAR activation in epididymal white adipose tissue (Muenzner et al., 2013)decreased liver retinyl ester levels (Muenzner et al., 2013)glucose tolerance not impaired (Muenzner et al., 2013)
Mouse	Adipocyte-specific overexpression of human RBP4	increased RBP4 protein levels in adipose tissue (Lee et al., 2016)circulating RBP4 and retinol levels unaltered when fed normal chow, increased when fed high-fat diet (Lee et al., 2016)glucose intolerance and hepatic steatosis (Lee et al., 2016)elevated concentrations of non-esterified fatty acids in plasma (Lee et al., 2016)increased weight gain when feeding a high-fat diet (Lee et al., 2016)elevated non-esterified fatty acid uptake and increased gluconeogenic gene expression in liver (Lee et al., 2016)signs of increased visceral adipose tissue inflammation and altered retinoid homeostasis in liver (Lee et al., 2016)
Mouse	Hepatocyte-specific knockout of RBP4	RBP4 undetectable and retinol reduced by more than 93% in serum (Thompson et al., 2017)hepatic retinol and retinyl ester levels unchanged (Thompson et al., 2017)moderately increased body weights, weight gain, and fat mass when fed a high-fat/high-sucrose diet (Thompson et al., 2017)no alterations in insulin sensitivity or glucose tolerance on control or high-fat/high-sucrose diet (Thompson et al., 2017)
Mouse	Human *RBP4* open reading frame in the mouse *RBP4* locus	rescue of plasma RBP4 levels, of retinol levels and retinal function when crossed into RBP4-deficient mice (Liu et al., 2017), analyzed after backcrossing to C57BL/6 genetic background
Mouse	Long-term liver-specific overexpression of murine RBP4	increased serum RBP4 and retinol levels (Fedders et al., 2018)liver retinyl ester levels unchanged (Fedders et al., 2018)insulin response and glucose tolerance not impaired on either normal chow or high-fat diet (Fedders et al., 2018)
Human	Compound heterozygous p.I59N and p.G93D	Night blindness (Biesalski et al., 1999; Seeliger et al., 1999)modest retinal dystrophy (Biesalski et al., 1999; Seeliger et al., 1999)undetectable RBP4 and reduced retinol concentrations in serum (Seeliger et al., 1999)
Human	Homozygous c.111 + 1G > A	Retinal dystrophy (Cukras et al., 2012)developmental abnormalities (Cukras et al., 2012)undetectable RBP4 and reduced retinol concentrations in serum (Cukras et al., 2012)
Human	Heterozygous p.A73T and p.A75T	Causing autosomal dominant congenital eye malformations (incl. microphthalmia, anophthalmia, and coloboma disease) (Chou et al., 2015)poor binding of mutated RBP4 to retinol but higher affinity to STRA6 (Chou et al., 2015)
Human	Bi-allelic c.248 + 1G > A	Retinal dystrophy (Khan et al., 2017)ocular coloboma (Khan et al., 2017)undetectable RBP4 levels in serum (Khan et al., 2017)
Human	Homozygous c.67 C > T	Retinitis pigmentosa and childhood acne vulgaris (Cehajic-Kapetanovic et al., 2020)

## Author Contributions

JSS and MS wrote the manuscript. AL gave intellectual input and edited the manuscript. All authors contributed to the article and approved the submitted version.

## Conflict of Interest

The authors declare that the research was conducted in the absence of any commercial or financial relationships that could be construed as a potential conflict of interest.
